# Attitudes towards deprescribing and the influence of health literacy among older Australians

**DOI:** 10.1017/S1463423618000919

**Published:** 2019-06-20

**Authors:** Robyn Gillespie, Judy Mullan, Lindsey Harrison

**Affiliations:** 1 School of Medicine, University of Wollongong, Wollongong, NSW, Australia; 2 Centre for Health Research Illawarra Shoalhaven Population (CHRISP), Australian Health Services Research Institute, University of Wollongong, Wollongong, NSW, Australia; 3 School of Health and Society, University of Wollongong, Wollongong, NSW, Australia

**Keywords:** deprescribing, health literacy, older adults, polypharmacy, primary care, quantitative analysis

## Abstract

**Aim:**

This study aimed to explore attitudes, beliefs and experiences regarding polypharmacy and discontinuing medications, or deprescribing, among community living older adults aged ≥65 years, using ≥5 medications. It also aimed to investigate if health literacy capabilities influenced attitudes and beliefs towards deprescribing.

**Background:**

Polypharmacy use is common among Australian older adults. However, little is known about their attitudes towards polypharmacy use or towards stopping medications. Previous studies indicate that health literacy levels tend to be lower in older adults, resulting in poor knowledge about medications.

**Methods:**

A self-administered survey was conducted using two previously validated tools; the Patients’ Attitude Towards Deprescribing (PATD) tool to measure attitudes towards polypharmacy use and deprescribing and the All Aspects of Health Literacy Scale (AAHLS) to measure functional, communicative and critical health literacy. Descriptive statistical analysis was conducted.

**Findings:**

The 137 responses showed that 80% thought all their medications were necessary and were comfortable with the number taken. Wanting to reduce the number of medications taken was associated with concerns about the amount taken (*P*<0.001), experiencing side effects (*P*<0.001), or believing that one or more medications were no longer needed (*P*<0.000). Those who were using ten or more medications were more likely to want to reduce the number taken (*P*=0.019). Most (88%) respondents would be willing to stop medication/s in the context of receiving this advice from their doctor. Willingness to consider stopping correlated with higher scores on the critical health literacy subscale (*P*<0.021) and overall AAHLS score (*P*<0.009). Those with higher scores on the overall AAHLS measure were more likely to report that they understood why their medications were prescribed (*P*<0.000) and were more likely to participate in decision-making (*P*=0.027). Opportunities to proactively consider deprescribing may be missed, as one third of the respondents could not recall a recent review of their medications.

## Introduction

Worldwide, the number of older adults is increasing rapidly and in high income countries between 20 and 30% of the population are aged over 60 (World Health Organization, 2015). While the health of older adults varies, the prevalence of multiple morbidities among them is markedly higher (Marengoni *et al*., 2011), which in-part contributes to increasing polypharmacy (Hovstadius *et al*., 2010; Guthrie *et al*., 2015; Kantor *et al*., 2015). In older adults, polypharmacy, commonly defined as taking five or more concurrent medications (Gnjidic, 2012) is potentially problematic, because it can be associated with an increased risk of falls (Niikawa *et al*., 2017), mortality (Jyrkkä *et al*., 2009b) and hospitalisations (Reason *et al*., 2012). Deprescribing, a strategy designed to reduce inappropriate polypharmacy, involves discontinuing medications that are no longer required or are potentially harmful (Page *et al*., 2018). Non-randomised trials of deprescribing in older adults using polypharmacy have shown a sustained decrease in the number of medications taken and an improvement in clinical outcomes (Page *et al*., 2016; Garfinkel, 2018).

Deprescribing, however, may not be considered a routine part of the prescribing continuum (Bain *et al*., 2008) because of health system, prescriber and patient related barriers (Gillespie *et al*., 2018). Patient-related barriers include; beliefs about the necessity of medications, fear of symptom return or withdrawal symptoms (Reeve *et al*., 2013b). Other patient-related barriers may include an inability to comprehend health information or to communicate their preferences with their doctors (Holmes and Todd, 2017). Older age has been associated with low health literacy (Baker *et al*., 2000; Kutner *et al*., 2006; Wolf *et al*., 2010; Bostock and Steptoe, 2012; Geboers *et al*., 2016) which contributes to poor knowledge and understanding about medications (Mosher *et al*., 2012) and polypharmacy (Huxhagen, 2018).

Worldwide, studies indicate that most patients are hypothetically willing to stop one or more of their regular medications (71–93%), if their doctor thought it was possible (Galazzi *et al*., 2016; Ng *et al*., 2017; Sirois *et al*., 2017; Tegegn *et al*., 2018). These findings concur with three Australian studies which found that patients’ willingness to consider deprescribing ranged from 79–92% (Reeve *et al*., 2013c; Qi *et al*., 2015; Kalogianis *et al*., 2016). These studies investigated interest towards deprescribing for older aged care facility residents (Kalogianis *et al*., 2016), older hospital inpatients (Qi *et al*., 2015) and adults (≥18 years) with multiple chronic morbidities (Reeve *et al*., 2013c). They did not, however, investigate the attitudes towards deprescribing of autonomous community living older adults, who make up the majority of the older Australian population (Australian Institute of Health and Welfare, 2017), and who are likely to use polypharmacy (Morgan *et al*., 2012).

This study aimed to explore attitudes, beliefs and experiences regarding polypharmacy and deprescribing among community-living older adults, taking five or more medications. It also aimed to investigate if health literacy capabilities influenced older adults’ attitudes and beliefs towards deprescribing.

## Methods

### Study population and recruitment

Independent community living older adults, aged 65 years or older, taking five or more prescribed medications, were invited to complete an anonymous survey between October 2015 and November 2016. Two different approaches were used to recruit participants. The first involved a purposive sample of 23 community pharmacies, located in regional Australia, which were invited to assist with distributing surveys to eligible participants. Eleven pharmacies volunteered to assist and were provided with a total of 330 surveys for distribution. However, it is not known whether all the surveys were distributed. The second approach involved 415 surveys being distributed by the lead author to eligible participants attending older adult community groups located within the same regional area. Only those who were literate in English were able to complete the survey, which they returned via reply paid envelopes.

### Survey

The 42 item survey included: 15 demographic items; 13 Patients’ Attitude Towards Deprescribing (PATD) questionnaire items (Reeve *et al*., 2013a); 10 All Aspects of Health Literacy Scale (AAHLS) items (Chinn and McCarthy, 2013); three items from the Canadian Survey of Experiences with Primary Health Care (Statistics Canada, 2009); and one additional item to measure the actual impact of cost. The original validated PATD questionnaire was chosen because it measures the medication users’ attitudes towards deprescribing and polypharmacy (Reeve *et al*., 2013a). However, two of the PATD questions were removed because they focus on attitudes towards pharmacist involvement and follow-up which was not the focus of this study. The AAHLS was chosen because it attempts to assess multiple dimensions of health literacy; functional communicative and critical health literacy skills (Chinn and McCarthy, 2013). These three domains are based on Nutbeam’s health literacy model which acknowledges the importance of a wider set of cognitive and social skills to enable individuals to interact with and interpret the health system and health information (Nutbeam, 2000). The functional health literacy items in AAHLS measure reading and writing skills and the ability to access support networks when reading and/or writing skills are limited. The communicative health literacy items measure information gathering and interactive skills required to consult with health practitioners. Critical health literacy items measure respondents’ information appraisal skills in assessing the relevance, reliability, credibility and validity of health information (Chinn, 2011; Chinn and McCarthy, 2013). For this study, three of the AAHLS questions which address health literacy capabilities at the level of community engagement were not included.

### Statistical analysis

Descriptive statistics were used to analyse the data using SPSS version 24 (IMB Corp, 2016). Frequencies and percentages were reported for responses to the PATD questions 1–10 and the additional questions 11–17. For the AAHLS potential scores for each individual item ranged from 1 (lowest) to 3 (highest) (Chinn and McCarthy, 2013). Items that were negatively worded were reverse coded. Responses to each item were summed and a summed score was calculated for each subscale (potential functional scores 3–9; communicative scores 3–9; critical scores 4–12) and for the AAHLS as a whole (potential scores 10–30). Based on evidence (Chinn and McCarthy, 2013), no cut-off for adequate health literacy was determined using this scale. However, for the purposes of analysis, lower summed scores (for each subscale and the AAHLS as a whole) were assumed to indicate lower health literacy capabilities. For analysis purposes, age was grouped into younger (≤76 years) or older (77+); Socio-Economic Index For Areas (SEIFA) was divided into high or low (Australian Bureau of Statistics, 2013); and the number of medications were grouped into polypharmacy (5–9 medications) and excessive polypharmacy (≥10 medications) (Jyrkkä *et al*., 2009a).

Spearman’s correlations were used to test for any associations between PATD items. Mann–Whitney *U* tests or Kruskal–Wallis H tests were used to investigate differences between groups of dichotomous or multinomial non-parametric ordinal items. Significant associations were assumed if the *P* value was ≤0.05.

## Results

A total of 187 respondents returned the survey, suggesting a 25.1% response rate if all surveys were distributed. This response rate compares well with evidence that 27.9% is an average response rate for surveys (Guo *et al*., 2016). Fifty survey responses were excluded from the final analysis because respondents were less than 65 years old, gave no indication of their age and/or number of prescribed medications, or were taking fewer than five prescription medications. Data quality was high. For the first 10 items of the PATD questions the median percentage of missing responses per item was 1.0% (range 0.0–4.4%) and for the AAHLS items the median percentage missing was 1.5% (range 0.7–5.1%).

Table 1 presents the demographic characteristics of the 137 respondents included in the analysis. The median age of respondents was 76 years which included more females (60.5%). Respondents were prescribed a median of seven medications and were living with a median of three self-reported diseases. Over two-thirds (66.4%) reported good to excellent health and most (76.6%) reported experiencing a good to excellent quality of life (QoL). The majority of respondents were either born in Australia (73%) or emigrated from English speaking countries (20.4%) and more than half (51.1%) had completed either an Apprenticeship/Trade or university qualification. Respondents resided in locations which were almost evenly distributed between high (48.9%) and low (50.4%) SEIFA, which is a measure of the level of socio-economic advantage and disadvantage of an area, relative to other areas (Australian Bureau of Statistics, 2013). Only one in five lived in inner regional locations, as defined by the Australian Statistical Geography Standard with the remainder (79.6%) living in a major city. No relevant significant associations were found between individual respondent characteristics (listed in Table 1) and willingness to stop medications (data not shown).

**Table 1:** Respondent characteristics

Characteristic	Respondent (*n*=137)
Age (years), median (IQR)	76 (73–83)
Gender	
Female	83 (60.5)
Male	52 (38.0)
Missing	2 (1.5)
Number of medications (median) (IQR)	7 ( 5–9)
Number of illness (self-report) (median) (IQR)	3 (2–4)
	Number of respondents (%)
Self-reported health status	
Very good or excellent	30 (21.9)
Good	61 (44.5)
Fair or poor	42 (30.7)
Missing	4 (2.9)
Self-reported QoL	
Very good or excellent	54 (39.4)
Good	51 (37.2)
Fair or poor	29 (21.2)
Missing	3 (2.2)
SEIFA decile	
High	67 (48.9)
Low	69 (50.4)
Missing	1 (0.7)
Location (ASGC)	
City	109 (79.6)
Inner regional	27 (19.7)
Missing	1 (0.7)
Highest education completed	
Primary or less	3 (2.2)
High school (year 10 or below)	48 (35.0)
High school (year 12 complete)	13 (9.5)
TAFE/Trade/Apprenticeship	41 (29.9)
University or higher	29 (21.2)
Missing	3 (2.2)
Country of birth	
Australia	100 (73)
UK/Ireland	25 (18.2)
Europe	5 (3.6)
N.Z.	3 (2.2)
Other	1 (0.7)
missing	3 (2.2)

IQR=interquartile range; QoL=quality of life; SEIFA=Socio-Economic Index for Areas; ASGC=Australian Statistical Geography Standard.

### 

#### Responses to the PATD questionnaire

**Figure 1:**
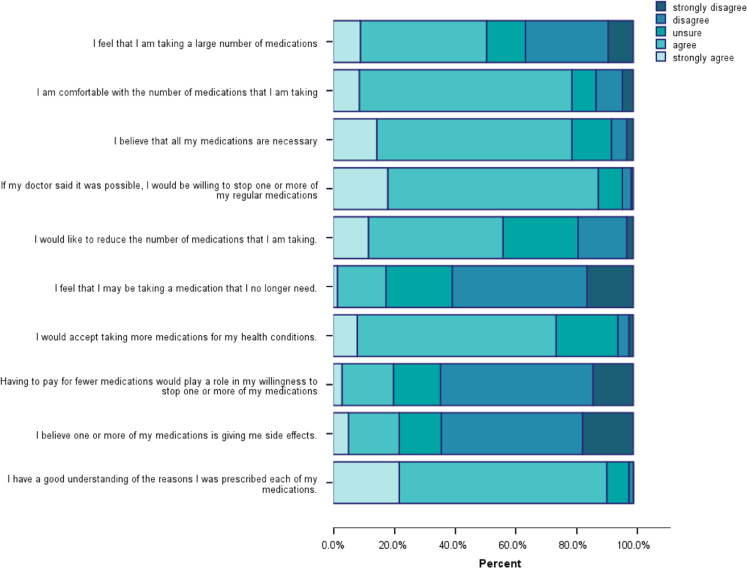
Patients’ Attitude Towards Deprescribing responses:questions 1–10.

The PATD responses (Figure 1) show that almost half of the respondents strongly agreed/agreed (49%) that they were taking a large number of medications. However, the majority of them were comfortable with the number of medications they were taking (80%) and believed they were necessary (80%). Nearly all respondents (91%) believed that they understood why they were taking their medications and most of them (74%) agreed that they would accept taking more medications for their health conditions.

Most respondents were unsure or disagreed (82%) that they were taking medications that they did not need and yet 88% agreed that they would be willing to stop one or more of their medications, if their doctor thought it possible. Importantly, over half (56%) agreed that they would like to reduce the number of medications they were taking. Approximately, one-fifth of the respondents (22%) agreed that their medications were giving them side effects and a similar proportion (20%) agreed that costs would impact on their willingness to stop one or more medications (Figure 1).

The results in Table 2 suggest that less than half of the respondents (45.3%) reported having actually stopped a medication in the past. Perceptions of what constituted ‘a lot’ of medications varied, with almost one third (32.1%) considering 5–9 medications was ‘a lot’ and a greater proportion (41.6%) considering that 10–14 was ‘a lot’. The majority of respondents (62.7%) indicated that that they would be comfortable taking ≤eight medications per day.

**Table 2:** Patients’ Attitude Towards Deprescribing (PATD) responses (questions 11–13) and additional questions

	Answer	*N* (%)
PATD responses (questions 11–13)		
Have you ever tried to stop a regular medication		
	Yes	62 (45.3)
	No	74 (54.0)
	Missing	1 (0.7)
How many tablets/capsules taken each day would you consider to be a lot?		
	5–9	44 (32.1)
	10–14	57 (41.6)
	15–19	21 (15.3)
	>20	10 (7.3)
	Missing	5 (3.6)
What is the maximum number of tablets/capsules that you would be comfortable taking in one day? (Pictorial response options)		
	4	35 (25.5)
	8	51 (37.2)
	12	31 (22.6)
	16	5 (3.6)
	20	2 (1.5)
	24	2 (1.5)
	Missing	11 (8.0)
Additional questions 14–17		
In the past 12 months, have you delayed or not bought one or several medications because you needed to spend your money on other items?		
	Yes	4 (3.0)
	No	128 (93.4)
	Missing	5 (3.6)
In the past 12 months, has your doctor taken time during a consultation to check all the different medications you are using, including medication prescribed by other medical doctors?		
	Yes	90 (65.7)
	No	42 (30.7)
	Missing	5 (3.6)
In the past 12 months, how often did your doctor involve you in decisions related to your medications?		
	Rarely	21 (15.3)
	Sometimes	41 (30.0)
	Often	57 (41.6)
	N/A	10 (7.3)
	Missing	8 (5.8)
Does your doctor allow you enough time to discuss your feelings, fears or concerns about new medicines or medicines you may have been taking for some time?		
	Rarely	13 (9.5)
	Sometimes	39 (28.5)
	Often	73 (53.3)
	N/A	7 (5.1)
	Missing	5 (3.6)

N/A=not applicable.

The results of Table 2 also highlight that the majority of respondents (93.4%) did not find paying for medications a financial burden and most (65.7%) recalled their doctor checking their medication in the past 12 months. Just over half (53.3%) believed that their doctors often allowed for enough time to discuss their feelings, fears and concerns about their medications and only 15.3% believed that their doctor rarely involved them in decisions about their medication(s).

#### Association between number of medications taken and PATD items

Analysis was undertaken to investigate if there were any correlations between the number of medications taken (5–9 or ≥10 or more) and responses to the first ten PATD items (see Table 3). Those who were using ten or more were significantly more likely to want to reduce the number of medications taken, felt that they were taking a large number of medications, and that they were taking medications they no longer needed. They were also significantly more likely to feel that one or more of their medications were giving them side effects. In contrast, respondents in the group taking five to nine medications were significantly more likely to be comfortable with the number of medications they were taking, more likely to believe that all their medications were necessary and more likely to understand why they were taking their medications. While it was not statistically significant, all the respondents taking ten or more medications were willing to consider stopping one or more of their regular medications.

**Table 3:** Differences in responses to Patients’ Attitude Towards Deprescribing (PATD) items and polypharmacy status

PATD items	5–9 medications *N* (%) agreed/strongly agreed^b^	≥10 medications *N* (%) agreed/strongly agreed^b^	*P*-value
I feel that I am taking a large number of medications	46 (44.2)	21 (77.8)	*P*=0.005^a^
I am comfortable with the number of medications that I am taking	90 (84.1)	19 (63.3)	*P*=0.015^a^
I believe that all my medications are necessary	89 (83.2)	20 (66.7)	*P*=0.042^a^
If my doctor said it was possible, I would be willing to stop one or more of my regular medications	91 (85.0)	30 (100.0)	*P*=0.062
I would like to reduce the number of medications that I am taking	56 (52.3)	21 (72.4)	*P*=0.019^a^
I feel that I may be taking one or more medications that I no longer need	13 (12.1)	11 (37.9)	*P*=0.008^a^
I would accept taking more medications for my health conditions	78 (73.6)	23 (76.7)	*P*=0.350
I have a good understanding of the reasons I was prescribed each of my medications	101 (95.3)	23 (76.7)	*P*=0.003^a^
Having to pay for fewer medications would play a role in my willingness to stop one or more of my medications	22 (21.0)	5 (17.2)	*P*=0.986
I believe one or more of my medications is giving me side effects	19 (17.9)	11 (36.7)	*P*=0.042^a^

a Mann–Whitney is significant at the *P*<0.05 level.

b Proportions are included to assist with the interpretation of the results.

#### Relationship between PATD items and the willingness to stop or reduce medications.

The current study investigated if there was an association between respondents’ willingness to stop one or more of their medications or their desire to reduce the number of their medication(s) and the first 10 PATD items (Table 4). There was a positive correlation between responses to wanting to reduce their medications, feeling that they were taking a large number of medications, taking a medication that is no longer needed and experiencing side effects. Negative correlations were noted between wanting to reduce medications and being comfortable with the number taken and believing that all current medications were necessary. When considering willingness to stop medication/s, the results highlight that there was a positive correlation between willingness to stop and a desire to reduce the number taken and also accepting more medications to manage health conditions. It should be noted that most correlations were weak with only one moderate correlation (*r* ^2^ 0.442) between wanting to reduce the number of medications and taking one or more medications that may no longer be needed (Table 4).

**Table 4:** Associations between Patients’ Attitude Towards Deprescribing (PATD) responses

PATD items	If my doctor said it was possible, I would be willing to stop one or more of my regular medications	I would like to reduce the number of medications that I am taking
I feel that I am taking a large number of medications	*r*_*s*_0.065, *P*<0.462	*r*_*s*_ 0.299, *P*<0.001*
I am comfortable with the number of medications that I am taking	*r*_*s*_ −0.004, *P*<0.966	*r*_*s*_ −0.335, *P*<0.000*
I believe that all my medications are necessary	*r*_*s*_ −0.003, *P*<0.977	*r*_*s*_ −0.256, *P*<0.003*
If my doctor said it was possible, I would be willing to stop one or more of my regular medications	–	*r*_*s*_0.194*, *P*<0.024
I would like to reduce the number of medications that I am taking	*r*_*s*_0.309, *P*<0.000*	–
I feel that I may be taking one or more medications that I no longer need	*r*_*s*_0.094, *P*<0.277	*r*_*s*_0.442, *P*<0.000*
I would accept taking more medications for my health conditions	*r*_*s*_0.194, *P*<0.024*	*r*_S_ −0.154, *P*<0.075
I have a good understanding of the reasons I was prescribed each of my medications	*r*_*s*_0.133, *P*<0.123	*r*_*s*_−0.076, *P*<0.378
Having to pay for fewer medications would play a role in my willingness to stop one or more of my medications	*r*_*s*_ −0.069, *P*<0.429	*r*_*s*_0.153, *P*<0.077
I believe one or more of my medications is giving me side effects	*r*_*s*_ −0.046, *P*<0.595	*r*_*s*_0.288, *P*<0.001*

*r* ^2^ Spearman correlation Interpretation of *r* values (Dancey and Reidy, 2007). Strength of relationship: ±0.1 to ±0.3 weak, ±0.4 to ±0.6 moderate, ±0.7 to±0.9, strong, ±1 perfect. *Spearman’s correlation is significant at the *P*<0.05 level.

#### Responses to the AAHLS questionnaire

**Figure 2:**
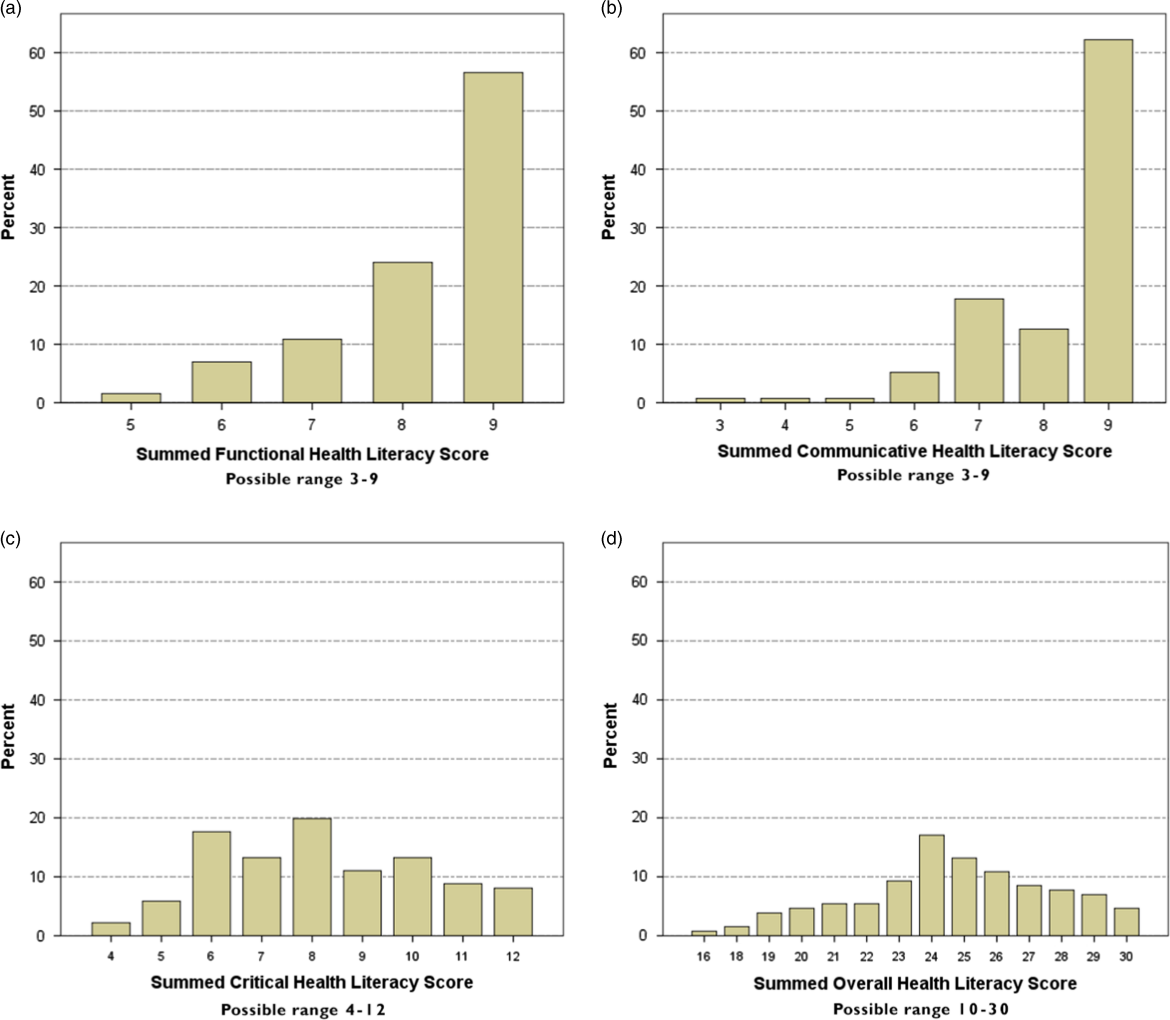
Distributions of health literacy scores. (a) Functional health literacy scores; (b) Communicative health literacy scores; (c) Critical health literacy scores; (d) Overall health literacy scores.

Most respondents demonstrated higher summed scores on both the functional (Figure 2a) and communicative subscales (Figure 2b), whereas summed critical health literacy subscale scores (Figure 2c) were more variable. Overall summed scores for the AAHLS (Figure 2d), as a whole, also indicated variability among respondents.

### Relationship between PATD items and summed health literacy scores

Table 5 highlights the significant correlations between health literacy scores and PATD items. Significant correlations were weak to moderate. There was a positive correlation between responses to having a good understanding of the reasons medications had been prescribed and each of the subscales of health literacy (functional, communicative, critical) and overall health literacy score. Further positive correlations between functional health literacy scores and being comfortable with the number of medications taken and believing that all medications were necessary were noted. In contrast, there was a negative correlation between both functional and communicative health literacy scores and feeling that that one or more medication/s may no longer be needed. Communicative health literacy scores were positively correlated with a willingness to accept taking more medications. In addition, there was a positive correlation between willingness to stop one or more medications and critical health literacy scores and overall AHHLS scores. A further positive correlation was noted between overall AHHLS scores and believing that all medications were necessary (Table 5).

**Table 5:** Relationship between Patients’ Attitude Towards Deprescribing (PATD) and summed health literacy scores

	Summed health literacy scores			
PATD items	Functional	Communicative	Critical	Overall
I feel that I am taking a large number of medications	*r*_s_ =−0.064, *P*<0.479	*r*_s_ =−0.030, *P*<0.735	*r*_s_ =0.083, *P*<0.345	*r*_s_ = 0.020, *P*<0.830
I am comfortable with the number of medications that I am taking	*r*_s_ = 0.206, *P*<0.019*	*r*_s_ = 0.037, *P*<0.671	*r*_s_ = −0.010, *P*<0.904	*r*_s_ = 0.095, *P*<0.286
I believe that all my medications are necessary	*r*_s_ = 0.268, *P*<0.002*	*r*_s_ = 0.152, *P*<0.078	*r*_s_ = 0.036, *P*<0.679	*r*_s_ = 0.182, *P*<0.039*
If my doctor said it was possible, I would be willing to stop one or more of my regular medications	*r*_s_ = 0.038, *P*<0.673	*r*_s_ = 0.164, *P*<0.057	*r*_s_ = 0.198, *P*<0.021*	*r*_s_ = 0.229, *P*<0.009*
I would like to reduce the number of medications that I am taking	*r*_s_ = −0.059, *p*<0.508	*r*_s_ = −0.009, *P*<0.916	*r*_s_ = 0.145, *P*<0.094	*r*_s_ = 0.093, *P*<0.294
I feel that I may be taking one or more medications that I no longer need	*r*_s_ =−0.244, *P*<0.005*	*r*_s_ = −0.266, *P*< 0.002*	*r*_s_ = −0.003, *P*<0.970	*r*_s_ = −0.156, *P*<0.078
I would accept taking more medications for my health conditions	*r*_s_ = 0.120, *P*<0.176	*r*_s_ = 0.316, *P*<0.000*	*r*_s_ = −0.156, *P*<0.070	*r*_s_ =−0.007, *P*<0.936
I have a good understanding of the reasons I was prescribed each of my medications	*r*_s_ = 0.336, *P*<0.000*	*r*_s_ =0.203, *P*<0.019*	*r*_s_ = 0.170, *P*<0.048*	*r*_s_ = 0.324, *P*<0.000*
Having to pay for fewer medications would play a role in my willingness to stop one or more of my medications	*r*_s_ = −0.118, *P*<0.186	*r*_s_ = −0.146, *P*<0.094	*r*_s_ =−0.003, *P*<0.973	*r*_s_ = −0.069, *P*<0.443
I believe one or more of my medications is giving me side effects	*r*_s_ = −0.031, *P*<0.731	*r*_s_ = −0.130, *P*<0.134	*r*_s_ =−0.050, *P*<0.561	*r*_s_ = −0.071, *P*<0.426

Spearman correlation. Interpretation of *r* values (Dancey and Reidy, 2007). Strength of relationship: ±0.1 to ±0.3 weak, ±0.4 to ±0.6 moderate, ±0.7 to ±0.9, strong, ±1 perfect. *Correlation is significant at the *p*<0.05 level.

Relationships between the PATD questions 11–13, the additional questions and summed overall AAHLS scores were investigated. Recalling a medication review (*U*= 1083.50, *P*=0.0031), believing there was enough time in consultations (*r*_*s*_ =196, *P*=0.031) and being involved in medication decision making (*r*_*s*_ =198, *P*=0.027) were associated with higher overall summed health literacy scores. There were no significant associations found with other items.

#### Individual characteristics and overall summed health literacy score.

Summed overall AAHLS scores were investigated to identify if there were any associations between health literacy capabilities and individual respondent characteristics. Respondents with lower overall AAHLS scores were significantly more likely to be in the group using 10 or more medications (*U*= 921.50, *P=*0.003) and were significantly more likely to self-report a poorer QoL (*χ* ^2^ (2)=7.241, *P*<0.027) and a poorer health status (*χ* ^2^ (2)=6.698, *P*<0.035). No other significant associations were found between overall AAHLS scores and respondent characteristics, such as gender, number of self-reported illnesses, location of residence, SEIFA decile and completed education levels.

## Discussion

The current study findings support previous evidence that most older adults are willing to consider stopping one or more of their medications if deemed appropriate by their doctor (Qi *et al*., 2015; Galazzi *et al*., 2016; Kalogianis *et al*., 2016; Sirois *et al*., 2017; Tegegn *et al*., 2018). These findings also concur with qualitative evidence indicating that older adults respect their doctor’s suggestions about deprescribing (Linsky *et al*., 2015a; Clyne *et al*., 2017), especially if they have established a long-term relationship and trust their doctor (Moen *et al*., 2009; Clyne *et al*., 2017). Potentially, trust and confidence in their doctors’ decisions may also explain why most respondents felt comfortable with taking their medications and thought they were all necessary.

It may be that specific cut-points to identify polypharmacy have limited usefulness in clinical practice (Cadogan *et al*., 2016). It is apparent that many older adults have a pragmatic approach to polypharmacy, in that most would consider taking more medications if their health required it and in practice, GPs also do not adhere to a fixed definition of polypharmacy (Moen *et al*., 2010; Linsky *et al*., 2015b). The emphasis should be on managing the ongoing appropriateness of medications for each individual, taking into account their preferences (Duerden and Payne, 2015) and considering the overall burden of polypharmacy use for the patient (Krska *et al*., 2017).

The burden of polypharmacy may be influential in wanting to stop medications. Our finding that respondents taking ten or more medications were more likely than those taking 5–9 to want to reduce the number of medications taken is novel, contradicting previous studies which found no significant relationship between the number taken and a desire to reduce (Galazzi *et al*., 2016; Kalogianis *et al*., 2016). This suggests that those taking fewer than ten medications may not think to discuss stopping any with their doctor because, as the findings show, they are comfortable with the number they are taking. In addition, some respondents were more likely to consider reducing the number of their medications, including those who were not comfortable with the number they were taking, believed they were taking medications that they no longer needed, or if they were experiencing side effects.

In the current study, one-fifth of the respondents indicated that reducing costs was a factor that would influence their willingness to consider stopping medication/s. This finding was unexpected as few respondents indicated that purchasing their medications was creating a financial burden. Furthermore, costs for prescription medications are subsidised by the Australian government, especially for older adults, many of whom would qualify for government healthcare subsidies. However, controlling costs may be an important issue in countries where prescription costs are not subsidised to the same extent (see The Commonwealth Fund 2017). For instance, a US qualitative study found that older adults, including those with health insurance, reported sometimes stopping their medication/s to help reduce costs (Elliott *et al*., 2007). As polypharmacy rates continue to increase, costs may become a greater burden. However, cost considerations should not be the basis on which deprescribing decisions are made.

Significant correlations between individual characteristics and health literacy levels were noted. Lower overall health literacy scores were associated with self-reports of poorer QoL and poorer overall health. These findings are consistent with previous research which found low health literacy levels to be an independent indicator for low self-rated health status among older adults (Bennett *et al*., 2009) and poorer QoL (Panagioti *et al*., 2018). It is not possible to determine from our results the direction of this relationship; poorer QoL and lower overall health may be contributing to lower health literacy scores. Definitions which regard health literacy as a dynamic skill that is changeable through the life course would support this interpretation (Squiers *et al*., 2012). As no significant correlations were noted between socio economic status and education in the present study, these factors do not appear to influence the health literacy scores in this sample. These findings are in contrast to other studies, which found that education status and indicators of socio economic position were associated with health literacy level among older adults (Bostock and Steptoe, 2012; Wolf *et al*., 2010).

Respondents scored highly on the functional health literacy scale which contradicted previous studies conducted among older adults in the United States using the Short Test of Functional Health Literacy in Adults (S-TOFLA) (Baker *et al*., 2000; Wolf *et al*., 2010) and in England using a brief four item scale (Bostock and Steptoe, 2012). Both these studies measure comprehension of text. Whereas the functional health literacy subscale in the AAHLS asks how often help is required to read health information or to fill in forms. Perhaps this difference explains the higher functional health literacy scores seen in the current study. To our knowledge, the AAHLS has not been used in another older adult population.

Despite the possible differences in functional health literacy measurement, it remains well known that functional health literacy is fundamental to being able to access and understand written medication information, such as prescription labels or Patient Information Leaflets (Wali *et al*., 2016). This was reflected in the current study with higher functional health literacy scores positively correlating with reporting a good understanding of the reasons for each medication and being less likely to report taking a medication that was perceived to be no longer needed. These findings highlight that written medicines information, given as a part of any prescribing/deprescribing process, should keep in mind the functional literacy needs of older adults (Mullen *et al*., 2018).

Most health literacy research assesses patients’ interactions with written material, even though verbal communication regarding medication information is the preferred option for many (Hamrosi *et al*., 2014). Higher communicative health literacy scores among the respondents were associated with having a good understanding of prescribed medications and being willing to accept more medications to manage health problems. In contrast, lower scores on this subscale were associated with taking a medication that was perceived to be no longer needed. These results support the evidence that actively involving patients in verbal communications about their medications is important to facilitate a patient centred approach (Street, 2017) and is key to understanding patients’ preferences and expectations regarding the appropriateness of their medications (Reeve *et al*., 2014).

The critical health literacy results found in the present study are difficult to interpret, which may in part be due to problems in defining and measuring the concept of critical health literacy (Chinn, 2011; Chinn and McCarthy, 2013). Nutbeam (2000) and Ishikawa *et al*. (2008) define critical health literacy as a distinct skill which implies measurability. However, others such as Rubinelli *et al*. (2009) define it as a capacity which would preclude objective measurement. The critical health literacy items in the AAHLS tool were based on Ishikawa’s scale (Ishikawa *et al*., 2008) and were designed as a screening tool (Chinn and McCarthy, 2013). As such, the results measure the extent to which respondents engage in critical appraisal of information and do not give any further insight into how, or indeed if, individuals apply this knowledge to make sense of decisions about their health. How older adults might use or apply critical health literacy skills when considering stopping medications therefore remains unknown and requires further research.

It is notable that higher overall AAHLS scores were positively correlated with understanding the reasons for medications being prescribed, participating in the decision making process and recalling a medication review. All of these factors are likely to be supportive of older adults’ engaging with their health care provider to discuss deprescribing as a possible medication management strategy. Earlier qualitative studies conducted with GP participants suggested that older adults had a poor understanding of their medications due to old age or a lack of education (Schuling *et al*., 2012; Linsky *et al*., 2015b). However, the current study results indicate that older adults hold a different opinion of their capabilities and this has implications for practice, supporting the current emphasis on incorporating shared decision making into the deprescribing processes (Reeve *et al*., 2014; Jansen *et al*., 2016). However, over 30% of the respondents perceived that there was a lack of time during consultations, that they were not included in the decision-making process and/or could not recall a medication review. These findings suggest that opportunities remain to improve engagement with older adults, restructuring appointment times to allow for discussion about medications concerns, offering regular structured medication reviews and inviting older adults to participate, if they so choose, in decision-making processes regarding their medications.

## Limitations

It is important to acknowledge, that the sample size for this study was limited, although comparable to other similar studies (Reeve *et al*., 2013c; Ng *et al*., 2017; Sirois *et al*., 2017). Also, respondents were drawn from one region in Australia which suggests the results may not be generalisable. It is possible, that the older adults who chose to respond to the survey already held strong opinions about the use of multiple medications and this may have biased the findings. Furthermore, as this survey was self-administered, older adults who had low literacy or were from non-English speaking backgrounds were not likely to participate. This may have contributed to the high health literacy scores, particularly on the functional health literacy subscale. In addition, the majority of the surveys were distributed to community groups, which means that older adults who were active community members may be over represented in the sample. This may also mean that the views of frailer and socially isolated older adults were not included in the responses. Similarly, the views of older adults from outer regional or remote areas of Australia may not be represented.

## Conclusions

Older adults who are using polypharmacy are generally comfortable with their medications and experience few concerns. However, they may express an interest in stopping one or more of their medications in order to reduce the number they are taking, especially those who are using ten or more. Costs, experiencing side effects, or believing that medication/s may be unnecessary may result in a desire to reduce the number of medications taken. Higher health literacy scores were associated with key aspects such as involvement in decision making, knowledge of medications and willingness to stop. Appropriate written and verbal communications about medications are therefore important to allow all older adults to understand and access the information they require to participate in medication management decisions. The ongoing appropriateness of medications may not be regularly assessed for all older patients, suggesting missed opportunities to discuss and plan deprescribing proactively.
